# Cultural differences in room size perception

**DOI:** 10.1371/journal.pone.0176115

**Published:** 2017-04-20

**Authors:** Aurelie Saulton, Heinrich H. Bülthoff, Stephan de la Rosa, Trevor J. Dodds

**Affiliations:** Human Perception, Cognition and Action, Max Planck Institute for Biological Cybernetics, Tübingen, Germany; Ecole Polytechnique Federale de Lausanne, SWITZERLAND

## Abstract

Cultural differences in spatial perception have been little investigated, which gives rise to the impression that spatial cognitive processes might be universal. Contrary to this idea, we demonstrate cultural differences in spatial volume perception of computer generated rooms between Germans and South Koreans. We used a psychophysical task in which participants had to judge whether a rectangular room was larger or smaller than a square room of reference. We systematically varied the room rectangularity (depth to width aspect ratio) and the viewpoint (middle of the short wall vs. long wall) from which the room was viewed. South Koreans were significantly less biased by room rectangularity and viewpoint than their German counterparts. These results are in line with previous notions of general cognitive processing strategies being more context dependent in East Asian societies than Western ones. We point to the necessity of considering culturally-specific cognitive processing strategies in visual spatial cognition research.

## Introduction

Investigating cultural variations in space perception is important for our understanding of basic spatial cognitive processes underlying spatial judgments as well as for the development of urban planning in multicultural environments (e.g. public transport and other indoor spaces). Biases in spatial perception, especially volume perception of indoor spaces, have mostly been studied in Western cultures [1–3]. The goal of this study is to understand whether culture can affect perception of indoor spaces such as room size perception. We especially want to see whether biases known to occur in volume estimations of rooms in Westerners from Germany [3] are also present for East Asian participants from South Korea.

### Biases in space perception

Multiple spatial tasks require the incorporation of contextual information to be performed accurately. This is especially the case for area or volume perception of spaces. In comparing objects or even rooms of different volume, all dimensions of the space need to be taken into consideration in order to make an accurate judgment about the size of the space. If one or more dimensions of the spatial volume are neglected by the perceiver in favour of one single salient dimension of the space (focusing on one aspect of the stimulus while ignoring contextual information), size judgments become ineluctably biased [3–5].

A famous bias in size estimates of figures and objects is the *elongation* or *centration bias* demonstrated in Piagetian experiments e.g. a tall/thin cylinder judged as containing more volume than an equivalent size short/broad cylinder [6]. This type of bias was further demonstrated with adults in marketing and psychological studies [4, 7]. Typically, subjects tend to judge more elongated objects as greater in size compared to less elongated shapes: rectangles are perceived as larger than squares of equal area [8, 9]. According to multiple authors [4–6, 10] this bias illustrates the fact that perceivers center or anchor their attention on one single salient dimension of the space e.g. the longest linear dimension.

Interestingly, similar biases were also found for larger spaces like rooms [1–3]. In a recent study [3], we tested German participants in comparative volume judgments of virtual rooms with constant height. Participants had to compare the volume (the overall size) of a rectangular room varying in width-to-depth aspect ratio (later referred to as room rectangularity) to a constant square room displayed on a computer screen. Subjects could look around the room from two different viewpoints: the middle of the long wall or the middle of the short wall of the room. When the ratio of the rooms changed in such a way that *depth* increases but *width decreases* in relation to the observer, participants were biased toward perceiving the space as larger. Hence, the bias measured in room size perception could potentially be explained by participants attributing more weight to the depth of the space (depth relative to viewer) than to other physical dimensions of the space. Other biases in room size perception can occur when manipulating the height of a room [1]. For instance, Oberfeld and Hecht [1] have shown that physically higher rooms made the room appear narrower. However, the perceived height of the space was not affected by physical changes in room width. Overall, those results suggest that biases in room size perception could be similar to the elongation biases reported in size estimates of geometric volumes.

In addition to the previously mentioned effects of geometrical shape on room size perception [2, 3] research in the field of architecture suggests that a room’s acoustic [11–13], surface colors/lightness [1, 14–16] and furnishing [17] could affect its perceived size. For instance, in the case of psychoacoustics, rooms associated with longer reverberation time are known to elicit larger room size judgments [13]. While multiple studies have focused on the effect of psychophysical properties on the perceived spaciousness of the room, very little is known about how culture can affect perceptual judgments of room size. To investigate this aspect further, the present study compared room size perception between German and South Korean subjects using the exact same paradigm as in Saulton et al. (2016) [3]. Would similar room size biases be present in populations from a different culture?

### Cultural differences in perceptual judgment tasks

Through the past few years, evidence comparing Western (e.g. Europeans or North Americans) to non-Western populations, especially East Asian societies (e.g. Japan, China, South Korea) suggests that differences in cognitive style of thought could lead to differences in perceptual judgments [18–23].

In particular, it has been suggested that East Asians are more likely to process information holistically i.e. attending to the entire field and relations among objects (also referred to as field dependent [23]), whereas westerners are more prone to using analytic cognitive processes (also referred to as field independent [23]) i.e. focusing more on a salient stimulus feature independently of its surrounding spatial or social context (for review see: [22, 24]). Although differences in cognitive processing can be primed by certain experimental manipulations [21] and sometimes be present across individuals of the same culture [25], context dependent judgments are generally more prominent in East Asian populations than Western populations [22, 26].

This holistic vs analytic cognitive distinction between East Asians and Westerners was demonstrated with different populations in a variety of perceptual tasks involving language characteristics (Stroop interferences between verbal content and emotional tone [27]), social cognition [28], facial emotions [29], memory (scene recognition see: [30]) attention [31, 32] but also spatial reasoning tasks [19, 20].

An interesting example of spatial reasoning tasks showing differences in responses between East Asians and Westerners are perceptual spatial tasks involving numerosity judgments [21]. In Krishna et al. (2008), subjects had to estimate how many dots were represented on two types of line configuration (unidirectional vs. bidirectional path) containing the same number of dots but with different distances between the two endpoints of each line. Chinese participants were much less affected by the change in path configuration than North Americans who considered the path with the greatest distance between end points to contain more dots. In contrast, Chinese participants considered the number of dots to be relatively similar between the two lines. In other words, Chinese participants were less prone to the distance spatial bias than North American participants [21].

Similar results could occur in the case of room size perception: participants from East Asian culture might be less sensitive to spatial biases in estimating the volume of rooms than their German counterparts. To investigate this question, we chose to compare German perceptual judgments of virtual room size with those of South Korean participants. We previously found that German’s volume judgments were significantly biased by the egocentric depth of rooms which resulted in a significant interaction between the degree of room rectangularity and viewpoint: depending on viewpoints (middle of short wall vs. middle of long wall), rectangular rooms were either perceived as smaller or larger than square rooms of equal volume [3]. Here, we wanted to compare these data from German participants to South Korean judgments using the exact same visual volume comparison task [3]. In line with previous work showing less susceptibility to spatial biases in East Asian populations, we would expect South Korean participants to be significantly less biased by changes in room rectangularity and viewpoint than their German counterparts.

## Method

### Participants

36 students from Tübingen University, Germany (22 Males, 14 females; *Mean*_*Age*_ = 28.7; *SD* = 7.3) and 36 students from Seoul Korea University, South Korea (16 Males, 20 females, *Mean*_*Age*_ = 23; *SD* = 2.02) took part in the Experiment. All participants gave written informed consent prior to the study and were paid for their contribution. The research was reviewed and approved by the local Ethics Committee of the University of Tübingen “Ethik-Kommission an der Medizinischen Fakultät der Eberhard-Karls-Universität und am Universitätsklinikum Tübingen” and done according to the ethical guidelines required by the Max Planck Society “Ethikrats der Max Planck Gesellschaft”. Our investigation was conducted in accordance with the Declaration of Helsinki.

### Material and method

**Fig 1:**
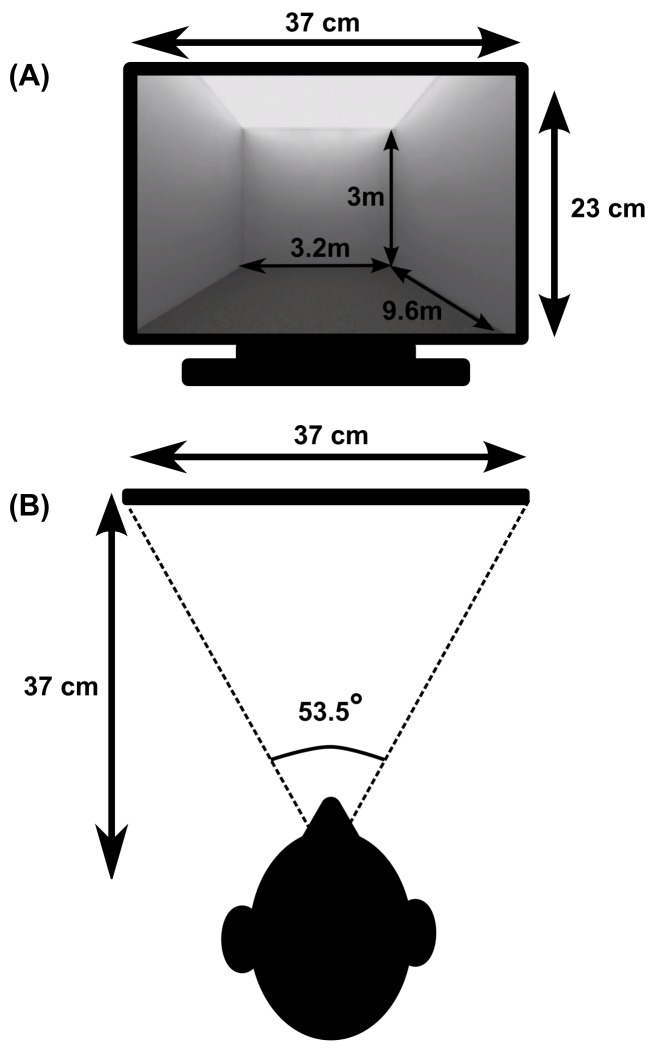
Geometrical relations between stimulus and observer. (A) Example of a rectangular stimulus shown from the middle of the short wall. The dimensions of the display and the 3D room are indicated via the arrows. Note that the depth dimension (9.6m) is not entirely visible from this displayed orientation: participants had to rotate their viewpoint to apprehend the entire room size using a joypad. (B) Plan view showing the observer in relation to the display. The geometrical field of view of the image displayed on the screen matched the observer’s field of view (53.5°), so that the computer screen acted as a “window” onto the virtual room, i.e. the displayed image is a 2D projection of what the participant would have seen if they were seated at the back wall of the virtual room.

German data are published in previous work [3] with no overlap with the present research question and analysis. New data were collected for South Korean participants. We used the exact same method and procedure as in previous work [3] (see Method of Experiment 1) to collect South Korean data. The study was conducted on a Macbook Pro 2,1 (Core 2 Duo T7600 @ 2.33GHz, 2 GB RAM and an ATI Mobility Radeon X1600 graphics processor with 256MB GDDR3 video memory) running at native 1680x1050 resolution on a 17” display. The geometric field of view was 53.5° horizontal and 35° vertical. Eye distance from the screen was about 37 cm. This corresponds to the distance for which the geometrical field of view of the image matched the visible field of the monitor (Fig 1). Not all depth cues (e.g. stereoscopic depth) were available in the current experiment. Specifically, we only provided pictorial depth cues, such as relative height, texture gradient, or linear perspective and motion parallax. The visualization and experiment workflow were implemented using the Unity Pro game engine (Version 3.5.4f1) running at a constant frame rate of 60 frames per second. Rooms displayed on the screen were rendered using perspective projection and presented with a ceiling (no windows and no furniture, see Fig 1). All rooms’ data were generated with Matlab R2011a 64 bit before the experiment.

### Procedure and design

**Fig 2:**
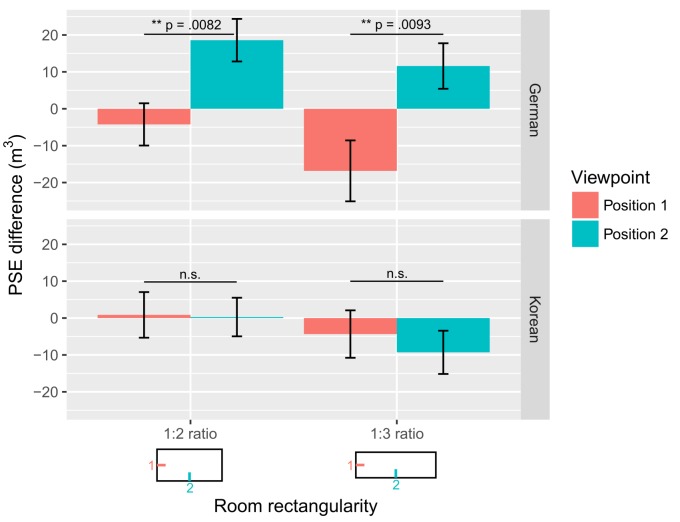
PSE differences for German and South Korean volume judgments of rectangular rooms of ratio 1:2 and 1:3 viewed from viewpoints 1 and 2. Viewpoints were located at the middle position of the short wall (position 1) or long wall (position 2) of the rooms. The y axis shows the difference between the PSE of rectangular and square rooms. PSE differences higher vs. lower than 0 mean volume judgments are biased, with rectangular rooms being judged as smaller vs. larger than a square room of equal volume. Error bars represent +- SE.

All instructions were translated from German by a native South Korean speaker. In both cultures, subjects were told to rely on their subjective impression of the overall size provided by the virtual space to express their volume judgment. Subjects wore noise cancelling headphones (with white noise) to help mask auditory influences. All subjects were seated against the back of the chair at a table frontally aligned with a screen (eye distance to the screen was about 37 cm). We did not use a chin rest to avoid discomfort. However, we instructed participants not to move their head as well as to maintain their direction of gaze towards the screen during the entire experiment. Participants were given a joypad to look around the space. The joypad rotated the viewpoint in yaw and pitch. The trial started with a randomly chosen viewing angle on the room (±4, ±8, ±12 degrees). The subject’s task was to compare a rectangular room (test stimulus) to a square room of reference (constant stimulus). The square room was always presented in the first interval. Subjects had 5 seconds to look around each room with a joypad. After visualizing the first room, a fixation cross appeared (2 seconds) and a second room appeared on the screen. After the room disappeared, subjects had to indicate whether the second room was larger or smaller than the first room. We manipulated the bias induced by the distance to the facing wall by changing the room rectangularity (width:depth aspect ratio from 1:1, to 1:2, to 1:3; the rooms’ height was kept at 3 meters) which participants viewed from two different viewpoints (Fig 2: position 1 or 2). Different groups of subjects were assigned to each viewpoint in the Korean and German cultures (*n* = 18 per viewpoint: note that two Korean subjects were excluded in each viewpoint for non-completion of the task). This was done to reduce the duration of the experiment (1 hour 30: including instruction time and 10 trials training). To generalize the effect to different room sizes, we varied the volume of the rectangular rooms from small spaces (min = 21 m^3^) to large spaces (max = 165 m^3^). Within each room aspect ratio category (1:1, 1:2, 1:3), 90 rooms were displayed, varying in their volume difference to the first reference room (constant volume of 93 m^3^). The volume differences between the first and second room were 0, ±9, ±18, ±45, ±72 m^3^. We presented 10 rooms within each of the 9 volume differences (90 in total). There was a total of 270 trials (3 aspect ratio categories multiplied by 90 rooms). The trials (ratios and rooms of different size) were presented in a random order. Subjects were given three breaks (of about 3 min) during the task (70, 140 and 210 trials). Sessions between breaks lasted about 18 min for Korean and German subjects.

### Statistics

For each participant and experimental condition, we used the method of constant stimuli and changed the room volume to determine the Point of Subjective Equality (PSE) for which the test room was perceived as equivalent to the reference room. We measured the PSE differences between rectangular and square room conditions.

We fit generalized linear models with the probit link function to obtain the PSE for each participant, room ratio and viewpoint [33] (similar methods were also used in related room size perception studies [34]). The model predictor, *x*, was the volume difference between the test room and reference room, and the outcome *y* either 0 (smaller) or 1 (larger). The PSE was the volume difference for which the predicted outcome “larger” was at 0.5 probability (stats package in R [35]).

Note that the constant stimulus was always presented in the first interval. The fixed presentation order could lead to a minor shift in PSE due to interval bias [36], e.g. participants preferring to report the room in the second interval as “larger” in case of uncertainty. However, the presentation order was the same for all conditions. Hence, we do not think that an interval bias would drastically change the interpretation of our results.

For the effect size, we reported generalized Eta square (*η*_*G*_^*2*^) in line with Bakeman’s recommendations [37]. The reader can also appreciate the details of the analysis by executing the R script provided in the supplementary material (S1 File).

## Results

### PSE differences across cultures

A participant’s PSE from a rectangular room condition minus their PSE from the square room condition (PSE difference) showed their bias due to rectangularity. For German participants, PSE differences for rectangular rooms varied across viewpoints from a mean of -4.24 m^3^ to 18.59 m^3^ for the 1:2 ratio (*SE* = 5.73 and 5.77), and from -16.84 m^3^ to 11.59 m^3^ for the 1:3 ratio (*SE* = 8.26 and 6.17). In contrast, Korean participants’ PSE differences were on average similar across viewpoints for the 1:2 ratio (0.86 m^3^ to 0.27 m^3^, *SE* = 6.18 and 5.23) and for the 1:3 ratio (mean of -4.34 m^3^ to -9.29 m^3^, *SE* = 6.43 and 5.86; Fig 2).

To directly compare results across the two cultures, we conducted an ANOVA on the PSE difference with culture and viewpoint as between-subject factors and room rectangularity as a within-subject factor. Importantly, our analysis showed the presence of a three-way interaction [*F*(1.66, 106.27) = 4.38, *p* = .020, *η*_*G*_^*2*^ = .028], which can be explained by a significant two-way interaction between *viewpoint and rectangularity* for Germans [*F*(1.72, 58.39) = 5.97, *p* = .0064, *η*_*G*_^*2*^ = .072] but not for Koreans [*F*(1.46, 43.76) = 0.27, *p* = .69, *η*_*G*_^*2*^ = .0034]. More specifically, German PSE difference was changing across viewpoint (smaller vs. larger) for each rectangularity [rooms of 1:2 ratio: *t*(34) = -2.81, *p* = .0082, *r* = .43; rooms of 1:3 ratio: *t*(34) = -2.76, *p* = .0093, *r* = .43; Fig 2]. In other words, the same rectangular rooms were perceived either larger or smaller than the square room of reference depending on the viewpoint: larger in position 1 and smaller in position 2. Hence, German volume judgments were *viewpoint dependent*. This was not the case for South Korean judgements, whose volume judgments did not significantly differ between viewpoints for each rectangular room [rooms of 1:2 ratio: *t*(30) = 0.072, *p* = .94, *r* = .013; rooms of 1:3 ratio: *t*(30) = 0.57, *p* = .57, *r* = .10; Fig 2]. Because one cannot infer from the null effect that volume judgments across viewpoints are similar for South Korean subjects, we determined the likelihood that the volume judgments measured in each viewpoint come from the null hypothesis distribution rather than from the alternative hypothesis distribution using Bayes factor (BayesFactor package in R [38]). The Bayesian analysis was calculated with a Jeffrey’s prior on the variance and a Cauchy prior on the standardized effect size, with a scale factor *r* = 0.71 for “medium” effect sizes [38, 39]. We found that viewpoint differences for Koreans were about 3 times more likely to come from the null than from the alternative hypothesis distribution (Bayes factor of 2.96 for rooms of 1:2 ratio and 2.62 for rooms of 1:3 ratio). Hence, Korean judgments appeared to be *viewpoint independent* and less biased by room rectangularity than German judgments.

### Differences in viewing patterns across cultures

**Fig 3:**
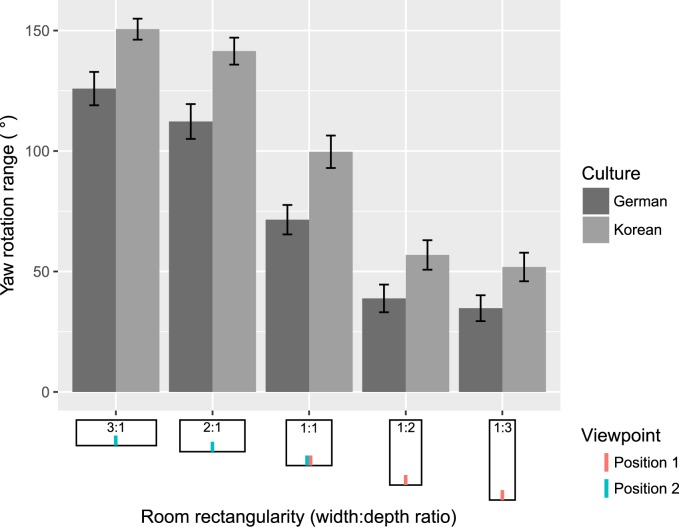
The yaw rotation range for each culture and each rectangularity. The rectangular rooms shown along the *x* axis depict the three room ratios (1:1, 1:2, 1:3) from the two viewpoints (position 1 and 2), giving a total of 5 ratios from the observer’s perspective. Error bars represent +- SE.

It has been suggested that differences in judgments between Westerners and East Asians arise from different viewing patterns [40]. Hence, one might expect Koreans to look around the space more than Germans (e.g. to incorporate as much information as possible about all rooms’ dimensions) explaining why they would be less biased than German participants by the rectangularity and viewpoint of the space. To investigate this aspect, we compared the range of yaw motion, i.e. left-right rotation of the virtual camera from viewpoint controlled via the joypad, used by South Koreans and Germans as a function of room rectangularity (Fig 3).

We analyzed the data using linear mixed-effect models with random intercepts indexed by subject. We chose linear models as a simple approximation of the relation of yaw rotation range and rectangularity, and the random intercepts account for the fact that some data come from the same subject. There was a significant main effect of *rectangularity* and a significant main effect of *culture* on the yaw rotation range with no interaction between the two factors [rectangularity: *χ*^*2*^(1) = 182.83, *p* < .001; culture: *χ*^*2*^(1) = 8.50, *p* = .0036]. While both cultures decreased their range of yaw rotation as the rooms became narrower from viewpoint, we noticed that Germans explored the space significantly less than South Koreans in all viewing conditions of the rooms (Fig 3). These results are consistent with the idea that South Koreans use more of the available visual information than Germans.

## Discussion

To summarize, we compared volume judgments of virtual rooms between South Koreans and Germans and found that Koreans were significantly less biased than Germans by room rectangularity and viewpoint. Those results are in line with previous studies showing that East Asians are less susceptible than Westerners to spatial biases present in tasks which require a judgment to be made in accordance with the surrounding context [21, 22].

Evidence from studies monitoring eye movements suggests that cultural differences in scene observation could arise from different viewing/attentional patterns [40]. Accordingly, we found hints that Koreans look around the rooms more than Germans (see section: *Differences in viewing patterns across cultures*). Making accurate volume judgments of rooms requires taking into account all size information associated to the different dimensions of the room [3–5]. Although we cannot directly prove that South Korean participants used all dimensions of the room to make their volume estimate, they clearly explored the space more than their German counterparts (by moving the joypad). This result could potentially explain why South Korean participants were less biased by a change in room rectangularity and viewpoint: Koreans might take into consideration a greater amount of information (e.g. information about the size of all dimensions of the room) than German participants whose results appeared to be predominantly biased by the egocentric depth of the space (for more details on this specific point, see: [3]). Further investigations are necessary to explore the relationship between viewing behavior and volume judgments.

Interestingly, Miyamoto et al. suggest that culturally specific patterns of attention or viewing behavior may be, at least partially, afforded by the perceptual environment [41]. For instance, Asian scenes (e.g. cities) generally contain more elements and are often more complex and ambiguous than Western scenes. It was shown that subjects primed by those complex and ambiguous Asian scenes were attending more to contextual information than subjects primed with Western scenes [41]. South Korean students involved in our study all grew up in Seoul or nearby megalopolis (this was not the case for German students, originated from relatively small German cities). This long term Asian urban surrounding might have encouraged a more contextual perception of the visual space for South Korean subjects compared to German subjects.

Several other suggestions have been put forward in the literature to explain performance differences in spatial tasks across people from different cultural backgrounds. For instance, studies suggest that navigation experience can positively affect spatial skills [42–44]. In populations with great navigational demands (Twe and Tjimba from Northern Namibia), it was shown that men who traveled more and further than others had better performance in spatial tasks (e.g. mental rotations [44]). Both groups of participants chosen in our study came from urban industrialized societies. Hence, we do not think the motives and modes of travel between South Korean and German significantly differ as to affect their spatial performance. Navigation experience is however not excluded from contributing to differences in volume perception tasks and would deserve further investigations.

Other reports indicate differences in spatial reasoning tasks between societies with languages relying on egocentric frames of reference (e.g. Indo-European languages) and societies based on allocentric spatial reference frames (e.g. Tzeltal from Mexico or Balinese from Indonesia, for review see: [45]). A language predominantly based on egocentric constructs like English or German would favor relative positioning of objects to the self (the man is on the left side of the tree) while languages favoring allocentric reference frame might either favor a geocentric system based on cardinal directions (the man is west of the tree) or object centered approaches (the man is behind the tree). In spatial pointing tasks, it was found that subjects judged the positions of arrows in line with the spatial reference frame privileged in their languages [45–47].

In our study, volume judgments of rooms were egocentric for Germans (volume judgments dependent on viewpoint) and allocentric for South Koreans (volume judgments independent of viewpoint). To our knowledge, the Korean language does not use cardinal directions or object centered approaches in usual conversations and also relies on an egocentric frame of reference. Hence, we do not think the cultural differences shown in this room size perception task reflect specific spatial-marking system associated with each language. Other linguistic variations related to spatial cognition might nevertheless exist between South Korean and German languages. However, evidence in favor of direct linguistic influence on spatial task interpretation remain debated [18, 47].

Overall, the exact causes underlying cultural differences in perceptual tasks are not well understood. We have discussed several hypotheses susceptible to explain the differential results obtained in our room size perception study between South Korean and German participants, including linguistic, navigation and viewing pattern differences. The concept of culture encompasses many factors which potentially contribute to the differences in volume perception between Germans and Koreans. Further investigations are needed to determine the exact reasons for the cultural differences in room size perception. This study constitutes a first step towards a better understanding of visual volume perception of indoor spaces and shows for the first time that room size judgments are, at least in part, culturally dependent.

## Conclusion

The goal of this research was to investigate whether spatial biases demonstrated in room size perception for Westerners from Germany would be less present in East Asian populations from South Korea. Here, we demonstrated for the first time that South Koreans are less biased than Germans by changes in room rectangularity and viewpoint. Although the mechanisms underlying these cultural differences are still unclear, we think this result is important to improve our general understanding of basic mechanisms involved in visual spatial perception. Those cultural differences are especially worth investigating to improve existing models for volume perception of spaces (e.g. [3]) and to make predictions for how to optimize physical space in a culturally sensitive manner (e.g. space stations).

## Supporting information

S1 File Data and analysis script. (ZIP)

## References

[1] Oberfeld D., & Hecht H. (2011). Fashion Versus Perception: The Impact of Surface Lightness on the Perceived Dimensions of Interior Space. Human Factors, 53(3), 284–298. doi: 10.1177/0018720811407331 21830513 10.1177/0018720811407331

[2] Sadalla E. K., & Oxley D. (1984). The Perception of Room Size: The Rectangularity Illusion. Environment and Behavior, 16(3), 394–405.

[3] Saulton A., Mohler B., Bülthoff H. H., & Dodds T. J. (2016). Egocentric biases in comparative volume judgments of rooms. Journal of Vision, 16(6):2, 1–16. doi: 10.1167/16.6.2 27049506 10.1167/16.6.2

[4] Krider R. E., Raghubir P., & Krishna A. (2001). Pizzas: π or Square? Psychophysical Biases in Area Comparisons. Marketing Science, 20(4), 405–425.

[5] Raghubir P., & Krishna A. (1999). Vital Dimensions in Volume Perception: Can the Eye Fool the Stomach? Journal of Marketing Research, 36(3), 313–326.

[6] Piaget J., & Inhelder B. (1969). The Psychology Of The Child. Basic Books.

[7] Anastasi A. (1936). The Estimation of Area. The Journal of General Psychology, 14(1), 201–225.

[8] Holmberg L., & Holmberg I. (1969). The perception of the area of rectangles as a function of the ratio between height and width Psychological Research Bulletin, 9. Lund: Lund University.

[9] Verge C. G., & Bogartz R. S. (1978). A functional measurement analysis of the development of dimensional coordination in children. Journal of Experimental Child Psychology, 25(2), 337–353. 660093 10.1016/0022-0965(78)90087-5

[10] Holmberg L. (1975). The Influence of Elongation on the Perception of Volume of Geometrically Simple Objects. Psychological Research Bulletin, 15, 1–18.

[11] Hameed, S., Pakarinen, J., Valde, K., & Pulkki, V. (2004). Psychoacoustic Cues in Room Size Perception. Presented at the Audio Engineering Society Convention 116, Audio Engineering Society. Retrieved from http://www.aes.org/e-lib/browse.cfm?elib=12755

[12] Larsson, P., Västfjäll, D., & Kleiner, M. (2002). Auditory-visual interaction in real and virtual rooms. In Proceedings of the Forum Acusticum, 3rd EAA European Congress on Acoustics, Sevilla, Spain. Retrieved from http://webistem.com/acoustics2008/acoustics2008/cd1/data/fa2002-sevilla/forumacusticum/archivos/psy05004.pdf

[13] Mershon D. H., Ballenger W. L., Little A. D., McMurtry P. L., & Buchanan J. L. (1989). Effects of room reflectance and background noise on perceived auditory distance. Perception, 18(3), 403–416. doi: 10.1068/p180403 2798023 10.1068/p180403

[14] Durak A., Camgöz Olguntürk N., Yener C., Güvenç D., & Gürçınar Y. (2007). Impact of lighting arrangements and illuminances on different impressions of a room. Building and Environment, 42(10), 3476–3482.

[15] Inui M., & Miyata T. (1973). Spaciousness in interiors. Lighting Research and Technology, 5(2), 103–111.

[16] Kwallek N. (1996). Office wall color: an assessment of spaciousness and preference. Perceptual and Motor Skills, 83(1), 49–50.

[17] von Castell C., Oberfeld D., & Hecht H. (2014). The Effect of Furnishing on Perceived Spatial Dimensions and Spaciousness of Interior Space. PLOS ONE, 9(11), e113267 doi: 10.1371/journal.pone.0113267 25409456 10.1371/journal.pone.0113267 PMC4237397

[18] Henrich J., Heine S. J., & Norenzayan A. (2010). The weirdest people in the world? The Behavioral and Brain Sciences, 33(2–3), 61-83-135. 10.1017/S0140525X0999152X 20550733

[19] Ji L.-J., Peng K., & Nisbett R. E. (2000). Culture, control, and perception of relationships in the environment. Journal of Personality and Social Psychology, 78(5), 943–955. 10821200 10.1037//0022-3514.78.5.943

[20] Kitayama S., Duffy S., Kawamura T., & Larsen J. T. (2003). Perceiving an object and its context in different cultures: a cultural look at new look. Psychological Science, 14(3), 201–206. doi: 10.1111/1467-9280.02432 12741741 10.1111/1467-9280.02432

[21] Krishna A., Zhou R., & Zhang S. (2008). The Effect of Self-Construal on Spatial Judgments. Journal of Consumer Research, 35(2), 337–348.

[22] Nisbett R. E., & Miyamoto Y. (2005). The influence of culture: holistic versus analytic perception. Trends in Cognitive Sciences, 9(10), 467–473. doi: 10.1016/j.tics.2005.08.004 16129648 10.1016/j.tics.2005.08.004

[23] Witkin H. A., & Berry J. W. (1975). Psychological Differentiation in Cross-Cultural Perspective. Journal of Cross-Cultural Psychology.

[24] Nisbett R. E., Peng K., Choi I., & Norenzayan A. (2001). Culture and systems of thought: Holistic versus analytic cognition. Psychological Review, 108(2), 291–310. 11381831 10.1037/0033-295x.108.2.291

[25] Choi I., Koo M., & Choi J. A. (2007). Individual differences in analytic versus holistic thinking. Personality & Social Psychology Bulletin, 33(5), 691–705. 17440200 10.1177/0146167206298568

[26] Norenzayan A., Smith E. E., Kim B. J., & Nisbett R. E. (2002). Cultural preferences for formal versus intuitive reasoning. Cognitive Science, 26(5), 653–684.

[27] Ishii K., Reyes J. A., & Kitayama S. (2003). Spontaneous attention to word content versus emotional tone: differences among three cultures. Psychological Science, 14(1), 39–46. doi: 10.1111/1467-9280.01416 12564752 10.1111/1467-9280.01416

[28] Miyamoto Y., & Kitayama S. (2002). Cultural variation in correspondence bias: The critical role of attitude diagnosticity of socially constrained behavior. Journal of Personality and Social Psychology, 83(5), 1239–1248. 12416925

[29] Masuda T., Ellsworth P. C., Mesquita B., Leu J., Tanida S., & Van de Veerdonk E. (2008). Placing the face in context: Cultural differences in the perception of facial emotion. Journal of Personality and Social Psychology, 94(3), 365–381. doi: 10.1037/0022-3514.94.3.365 18284287 10.1037/0022-3514.94.3.365

[30] Masuda T., & Nisbett R. E. (2001). Attending holistically versus analytically: comparing the context sensitivity of Japanese and Americans. Journal of Personality and Social Psychology, 81(5), 922–934. 11708567 10.1037//0022-3514.81.5.922

[31] Boduroglu A., Shah P., & Nisbett R. E. (2009). Cultural Differences in Allocation of Attention in Visual Information Processing. Journal of Cross-Cultural Psychology, 40(3), 349–360. doi: 10.1177/0022022108331005 20234851 10.1177/0022022108331005 PMC2838246

[32] Masuda T., & Nisbett R. E. (2006). Culture and Change Blindness. Cognitive Science, 30(2), 381–399. doi: 10.1207/s15516709cog0000_63 21702819 10.1207/s15516709cog0000_63

[33] McCullagh P., & Nelder J. A. (1989). Generalized linear models (2nd ed.). Boca Raton, FL: Chapman & Hall/CRC Press.

[34] von Castell C., Hecht H., & Oberfeld D. (2017). Measuring perceived ceiling height in a visual comparison task. Quarterly Journal of Experimental Psychology (2006), 70(3), 516–532. 10.1080/17470218.2015.1136658 26822335

[35] R Core Team (2016). R: A language and environment for statistical computing R Foundation for Statistical Computing, Vienna, Austria Retrieved from https://www.R-project.org/.

[36] Ulrich R., & Vorberg D. (2009). Estimating the difference limen in 2AFC tasks: pitfalls and improved estimators. Attention, Perception & Psychophysics, 71(6), 1219–1227. 10.3758/APP.71.6.1219 19633337

[37] Bakeman R. (2005). Recommended effect size statistics for repeated measures designs. Behavior Research Methods, 37(3), 379–384. 16405133 10.3758/bf03192707

[38] Morey, R. D., Rouder, J. N. (2015). BayesFactor: Computation of Bayes Factors for Common Designs. R package version 0.9.12–12. Retrieved from https://CRAN.R-project.org/package=BayesFactor

[39] Rouder J. N., Speckman P. L., Sun D., Morey R. D., & Iverson G. (2009). Bayesian t tests for accepting and rejecting the null hypothesis. Psychonomic Bulletin & Review, 16(2), 225–237. 19293088 10.3758/PBR.16.2.225

[40] Chua H. F., Boland J. E., & Nisbett R. E. (2005). Cultural variation in eye movements during scene perception. Proceedings of the National Academy of Sciences of the United States of America, 102(35), 12629–12633. doi: 10.1073/pnas.0506162102 16116075 10.1073/pnas.0506162102 PMC1194960

[41] Miyamoto Y., Nisbett R. E., & Masuda T. (2006). Culture and the physical environment. Holistic versus analytic perceptual affordances. Psychological Science, 17(2), 113–119. doi: 10.1111/j.1467-9280.2006.01673.x 16466418 10.1111/j.1467-9280.2006.01673.x

[42] Goeke C., Kornpetpanee S., Köster M., Fernández-Revelles A. B., Gramann K., & König P. (2015). Cultural background shapes spatial reference frame proclivity. Scientific Reports, 5, 11426 doi: 10.1038/srep11426 26073656 10.1038/srep11426 PMC4466779

[43] Padilla L. M., Creem-Regehr S. H., Stefanucci J. K., & Cashdan E. A. (2016). Sex differences in virtual navigation influenced by scale and navigation experience. Psychonomic Bulletin & Review, 1–9. 27714666 10.3758/s13423-016-1118-2

[44] Vashro L., & Cashdan E. (2015). Spatial cognition, mobility, and reproductive success in northwestern Namibia. Evolution and Human Behavior, 36(2), 123–129.

[45] Majid A., Bowerman M., Kita S., Haun D. B. M., & Levinson S. C. (2004). Can language restructure cognition? The case for space. Trends in Cognitive Sciences, 3(8), 108–114. 10.1016/j.tics.2004.01.003 15301750

[46] Levinson S. C. (2003). Space in Language and Cognition: Explorations in Cognitive Diversity. Cambridge University Press.

[47] Levinson S. C., Kita S., Haun D. B. M., & Rasch B. H. (2002). Returning the tables: language affects spatial reasoning. Cognition, 84(2), 155–188. 12175571 10.1016/s0010-0277(02)00045-8

